# Longitudinal associations of chemotherapy-induced peripheral neuropathy with psychological distress and health-related quality of life in colorectal cancer survivors: a prospective cohort study

**DOI:** 10.1007/s11764-025-01906-1

**Published:** 2025-11-21

**Authors:** N. D. Boekhoudt, F. Mols, C. Simons, N. R. Querido, S. O. Breukink, L. Valkenburg-van Iersel, M. L. G. Janssen-Heijnen, E. T. P. Keulen, M. P. Weijenberg, M. J. L. Bours

**Affiliations:** 1 Department of Epidemiology, GROW Research Institute for Oncology and Reproduction, Maastricht University, Maastricht, The Netherlands; 2 Department of Medical and Clinical Psychology, Tilburg University, Tilburg, The Netherlands; 3 Department of Surgery, GROW Research Institute for Oncology and Reproduction, NUTRIM Institute of Nutrition and Translational Research in Metabolism, Maastricht University Medical Centre+, Maastricht, The Netherlands; 4 Department of Internal Medicine, Division of Medical Oncology, GROW Research Institute for Oncology and Reproduction, Maastricht University Medical Centre+, Maastricht, The Netherlands; 5 Department of Clinical Epidemiology, VieCuri Medical Centre, Venlo, The Netherlands; 6 Department of Internal Medicine and Gastroenterology, Zuyderland Medical Centre Sittard-Geleen, Geleen, The Netherlands

**Keywords:** Colorectal cancer, Cancer survivors, Chemotherapy-induced peripheral neuropathy, Psychological distress, Health-related quality of life

## Abstract

**Purpose:**

Colorectal cancer (CRC) survivors receiving oxaliplatin-based chemotherapy frequently develop chemotherapy-induced peripheral neuropathy (CIPN). This study investigated the impact of CIPN symptoms on psychological distress and health-related quality of life (HRQoL) among oxaliplatin-treated CRC survivors up to 5 years post-treatment.

**Methods:**

124 CRC patients treated with oxaliplatin-based chemotherapy participating in a prospective cohort study were included in the longitudinal analyses. CIPN symptoms were measured at diagnosis (pre-treatment) and at 6 weeks and 6, 12, 24, and 60 months post-treatment using the European Organization for Research and Treatment of Cancer Quality of Life Questionnaire-CIPN20 (EORTC QLQ-CIPN20). Psychological distress, including anxiety and depressive symptoms, and HRQoL were measured at all post-treatment time points using the Hospital Anxiety and Depression Scale (HADS) and the EORTC QLQ-Core 30 (EORTC QLQ-C30), respectively. Confounder-adjusted longitudinal associations of CIPN symptoms with psychological distress and HRQoL were analysed with linear mixed regression models.

**Results:**

CIPN symptoms in oxaliplatin-treated CRC patients were highest at 6 weeks post-treatment, thereafter gradually decreasing over time. More severe CIPN symptoms were longitudinally associated with more psychological distress (HADS), including both higher anxiety and depressive symptoms, and with worse global health/QoL and physical and social functioning and more fatigue (EORTC QLQ-C30).

**Conclusion:**

CIPN symptoms, persisting over time among oxaliplatin-treated CRC survivors, were longitudinally associated with more psychological distress and lower HRQoL up to 5 years after chemotherapy treatment.

**Implications for Cancer Survivors:**

CIPN symptoms following oxaliplatin-based chemotherapy have a long-lasting impact on CRC survivors. Research on interventions aimed at mitigating CIPN symptoms is warranted.

**Supplementary Information:**

The online version contains supplementary material available at https://doi.org/10.1007/s11764-025-01906-1.

## Introduction

Chemotherapy-induced peripheral neuropathy (CIPN) is a common side-effect of chemotherapy, particularly in colorectal cancer (CRC) patients, limiting treatment dosage and negatively impacting health-related quality of life (HRQoL) [1, 2]. CIPN involves damage to the peripheral nerves, primarily in the hands and feet, causing symptoms that profoundly disrupt daily activities. These symptoms encompass both hyperactive sensory effects, such as hyperalgesia and allodynia characterized by heightened responses to stimuli, and hypoactive sensory effects, including numbness and reduced sensitivity to touch or pain [3]. Furthermore, patients may encounter challenges with fine motor skills and disturbances in vibratory and proprioceptive sensations, significantly hampering their ability to perform routine tasks such as buttoning a blouse, gripping a pen, or opening a bottle, due to sensations like pain, tingling, and weakness [3, 4]. A meta-analysis by Seretny et al. (2014) reported CIPN prevalence between 58–78% one month post-chemotherapy among mixed cancer types, including CRC [5]. In CRC patients treated with oxaliplatin-based regimens, prevalence of persisting CIPN symptoms was 42–73% at 6 months and 25–40% at 24 months after chemotherapy treatment [6].

CIPN is commonly linked to chemotherapy agents such as taxanes, platinum derivatives, vinca alkaloids, and proteasome inhibitors [7, 8]. Oxaliplatin is widely used in CRC treatment, being the primary adjuvant treatment option for stage III disease, administered to about two-thirds of stage III patients, and also an option for high-risk stage II patients [9, 10]. It works by forming interstrand DNA adducts that disrupt the cell cycle [11]. The severity of CIPN symptoms depends on the agent type, dosage, and any pre-existing nerve damage (e.g., related to diabetes) [7]. Although the peripheral nervous system can regenerate, recovery requires that nerve cell bodies remain intact and are not subject to further damage. In many cases, CIPN symptoms only partially resolve because of irreversible neural damage, indefinitely impacting HRQoL even after successful cancer treatment [7].

CIPN symptoms during chemotherapy are often managed by reducing the chemotherapy dose or stopping treatment, which can compromise treatment response [12, 13]. Beyond physical effects, CIPN also leads to fiscal strain. Patients with CIPN face 33% higher annual health care costs, and 25% greater work loss compared to those without CIPN [14]. A systematic review by Mols et al. (2014) showed that CIPN significantly reduces HRQoL in cancer patients [4]. However, few studies have examined the longitudinal impact that persisting CIPN symptoms after completing chemotherapy have on psychological distress (including both symptoms of anxiety and depression) and HRQoL, particularly in CRC survivors treated with oxaliplatin-based chemotherapy.

While CIPN has been shown to reduce HRQoL, informational gaps remain regarding long-lasting effects of post-treatment CIPN symptoms on CRC survivors, highlighting the need for further research. Therefore, this study aimed to investigate longitudinal associations of CIPN symptoms with psychological distress and HRQoL up to 5 years post-treatment among CRC survivors treated with oxaliplatin-based chemotherapy. Secondary aims included assessing whether CIPN severity differed between chemotherapy regimens (oxaliplatin-based versus non-oxaliplatin-based), differed according to sex and weight status of oxaliplatin-treated patients, and, to confirm the impact of chemotherapy treatment, between chemotherapy-treated and non-chemotherapy-treated patients who may report CIPN-like symptoms due to, for example, comorbidities. We hypothesized that CRC patients experiencing CIPN symptoms following oxaliplatin-based chemotherapy would report greater psychological distress and lower HRQoL up to 5 years after the end of treatment.

## Materials and methods

### Study design and population

Data were used from the Energy for life after ColoRectal cancer (EnCoRe) study, a multicenter prospective cohort study, initiated in 2012 [15], for which CRC patients were recruited at diagnosis in three hospitals in the province of Limburg in The Netherlands: Maastricht University Medical Centre+, Zuyderland Medical Centre, and VieCuri Medical Centre. Men and women, aged 18 years or older, diagnosed with stage I-III CRC were eligible for participation. Patients were excluded if they had stage IV CRC, lived outside of The Netherlands, could not read or speak Dutch, or had comorbidities obstructing participation (e.g., Alzheimer’s disease or sensory impairments). Participants were followed up from diagnosis until 60 months post-treatment, with study measurements at diagnosis before the start of treatment and at 6 weeks, and 6, 12, 24, and 60 months after the completion of treatment (surgery, chemotherapy, and/or radiotherapy). The EnCoRe study is approved by the Medical Ethical Committee of the University Hospital Maastricht and Maastricht University (NL38786.068.11) and is registered in the National Trial Register (NL6904) that is currently combined with the Research Portal of the Dutch Central Committee on Research Involving Human Subjects (CCMO, ID 25068). All participants signed informed consent upon participation. By October 1st 2021, 567 participants were included at diagnosis (43% participation rate). Our primary analyses were focused on a subsample of 124 participants at 6 weeks post-treatment who had received oxaliplatin-based chemotherapy, with follow-up data available from 111, 106, and 93 participants at 6, 12, and 24 months post-treatment, respectively (see Supplementary Fig. 1).

### Data collection

Data was collected by means of home visits performed by trained research assistants and dietitians at all time points, as described in more detail elsewhere [15]. CIPN, psychological distress, and HRQoL were assessed by means of validated patient-reported questionnaires, which were handed out on paper during home visits to be recollected and checked for completeness approximately 1 week later. In addition, detailed sociodemographic, clinical, and lifestyle data were collected by standardized questionnaires, from medical records, and by means of anthropometric measurements performed at participants’ homes.

#### Peripheral neuropathy

CIPN symptoms were assessed using the European Organization for Research and Treatment of Cancer Quality of Life Questionnaire-Chemotherapy-Induced Peripheral Neuropathy 20 (EORTC QLQ-CIPN20) [16], consisting of 20 items with response options on a 4-point Likert scale to assess the degree of CIPN symptoms over the past week. To determine the overall severity of CIPN symptoms, a total score was calculated as well as separate subscale scores covering sensory (9 items), motor (8 items), and autonomic (3 items) symptoms. Scores were transformed to a 0–100 point scale, with higher scores indicating more severe symptoms. Lavoie Smith et al. confirmed the questionnaire’s reliability and validity [17]. The EORTC QLQ-CIPN20 was administered to all study participants, regardless of whether they received chemotherapy, at diagnosis (pre-treatment) and at all post-treatment time points. The total CIPN score as well as the subscale scores were included in our analyses as independent (exposure) variables.

#### Psychological distress

Psychological distress was assessed using the Hospital Anxiety and Depression Scale (HADS), which consists of 14 items including 7 for anxiety and 7 for depressive symptoms [18]. The items can be answered on a 4-point Likert scale to assess the degree of symptoms experienced in the past week. An overall score for the level of psychological distress (0–42), and separate scores for the anxiety and depressive symptoms subscales (both ranging from 0–21) were calculated, with higher scores indicating more psychological distress or more anxiety and depressive symptoms. The HADS was shown to be valid and reliable for measuring symptoms of anxiety and depression in somatic, psychiatric, and primary care patients [19]. The HADS was administered at all post-treatment time points. Both the total HADS score and the two subscale scores were included in our analyses as dependent (outcome) variables.

#### Health-related quality of life

HRQoL was assessed using the 30-item EORTC QLQ-Core 30 (EORTC QLQ-C30), which is comprised of five functional scales (physical, role, cognitive, emotional, and social functioning), three symptom scales (fatigue, pain, and nausea and vomiting), and a global health/QoL scale [20]. Raw item scores were transformed into a 0–100 point scale, with higher scores indicating better functioning and global health/QoL, or worse symptoms over the past week. Reliability and validity of the EORTC QLQ-C30 were shown to be robust [21]. The EORTC QLQ-C30 was administered at all post-treatment time points. The global health/QoL and the physical, social, emotional, and cognitive functioning scales, as well as the fatigue symptom scale were included in our analyses as dependent (outcome) variables.

#### Sociodemographic, clinical, and lifestyle characteristics

Sociodemographic characteristics were assessed at diagnosis through self-report, including age (years), sex (men/women), and education level (low, medium, or high). Clinical characteristics were gathered from medical records and included tumor site (colon/rectum), cancer stage (I, II, or III), surgical treatment (yes/no), radiotherapy (yes/no), and chemotherapy (yes/no). In addition, chemotherapy treatment was subdivided according to its timing in relation to surgery, i.e., neo-adjuvant (pre-surgery) and adjuvant (post-surgery) chemotherapy; and according to the type of chemotherapy regimen, i.e., capecitabine + oxaliplatin (CAPOX), capecitabine monotherapy, 5-fluorouracil (5-FU) + oxaliplatin (FOLFOX), and else (hybrid treatments that in certain cases included oxaliplatin). Smoking status (never, former, or current) was self-reported at all time points. Alcohol intake (g/day) was derived from a detailed food frequency questionnaire at diagnosis, and from 7-day food diaries at post-treatment time points. Body mass index (BMI; kg/m2) was determined based on body weight (kg) measured at each time point and body height (m) measured at diagnosis. BMI categories were defined as underweight (BMI < 18.5 kg/m2), normal weight (BMI 18.5–24.9 kg/m2), overweight (BMI 25.0–29.9 kg/m2), and obese (BMI ≥ 30 kg/m2). The number of comorbidities (0, 1, or ≥ 2) was assessed at each time point using the 13-item Self-Administered Comorbidity Questionnaire [22].

### Statistical analyses

Descriptive statistics were employed to summarize patient characteristics at the 6-week post-treatment time point, which is the baseline for the longitudinal analyses. Categorical variables are presented as frequencies with percentages. Means with standard deviations (SD) are used for describing normally distributed continuous variables and medians with interquartile ranges (IQR) for non-normally distributed continuous variables.

To address the main research question, confounder-adjusted linear mixed-model regression analysis was performed to investigate longitudinal associations of CIPN symptoms with psychological distress and HRQoL from 6 weeks to 24 months post-treatment (primary analysis) and from 6 weeks to 60 months post-treatment (secondary analysis). A particular strength of linear mixed regression is that this modeling technique handles incomplete data very well, making optimal use of all available data from repeated measurements in study participants at the different post-treatment time points. Beta coefficients from the mixed regression models represent longitudinal associations, consisting of an intra-individual (within-subject) and inter-individual (between-subject) component [23]. Hybrid modeling was used to disentangle the intra-individual and inter-individual components [24]. Linear mixed regression models were adjusted for a priori selected confounders, including time since end of treatment (weeks), sex (men/women), age (years), smoking status (yes/no), alcohol intake (g/day), BMI (kg/m2), number of comorbidities (0, 1, or ≥ 2), tumor stage (I, II, or III), and CIPN score at diagnosis. Additionally, statistical interaction was analyzed by adding product terms to regression models for the following factors; time since end of treatment (weeks), sex (men/women), BMI (underweight/normal, or overweight/obese), and number of comorbidities (0–1, or ≥ 2). Regression coefficients, representing average change in the outcome score over time (i.e., HADS or EORTC QLQ-C30 score) for every 1-point increase in the exposure score (EORTC QLQ-CIPN20 score), were multiplied by 5 to better reflect a minimal clinically important difference (MCID) in the EORTC QLQ-CIPN20 score [25]. As a result, the regression coefficients indicate the average change in outcome scores over time associated with a clinically important 5-point change in the exposure score, adjusted for confounding.

To address secondary aims of this study, we performed confounder-adjusted linear mixed-model regression analysis to investigate the extent to which post-treatment CIPN symptom severity depends on the type of chemotherapy agent, and how post-treatment CIPN symptom severity differs over time between chemotherapy-treated patients and non-chemotherapy-treated patients who may report CIPN-like symptoms related to, e.g., comorbidities. These models were adjusted for tumor stage (I, II, or III) and for BMI (kg/m2), number of comorbidities (0, 1, or ≥ 2) and CIPN score at diagnosis. Regression coefficients indicate differences in the average change in CIPN scores over time between different treatment groups (i.e., oxaliplatin-based chemotherapy vs. other chemotherapy treatment, or chemotherapy treatment vs. no chemotherapy treatment). Tests for statistical interaction were conducted for BMI status at diagnosis (overweight/obese, yes/no) and time since end of treatment (weeks).

For all fully adjusted linear mixed regression models, a likelihood ratio test was used to assess whether adding a random slope improved the model fit. Statistical analyses were conducted using STATA 15.0 and IBM SPSS Statistics, version 28.0.1.0. P-values below 0.05 (two-sided) were deemed statistically significant.

## Results

### Patient characteristics

At 6 weeks post-treatment (i.e., the baseline for longitudinal analyses), 124 out of 504 participants had received oxaliplatin-based chemotherapy treatment (Supplementary Fig. 1). Baseline characteristics are summarized in Table 1. Oxaliplatin-treated patients were on average 63.6 years of age (SD: 7.7), including 65.3% men. There were 59.5% former smokers and 9.1% current smokers, and 31.4% never smoked. Median alcohol intake was 9.3 g/day among men and 1.7 g/day among women. Based on BMI categories, 28.2% of participants had normal weight, 39.5% overweight, and 32.3% were obese. Two or more comorbid conditions were reported by 42.3% of participants. Over two-thirds (68.5%) of participants were diagnosed with colon cancer, and 8.9% and 91.1% had stage II and III disease, respectively. CAPOX was the predominant oxaliplatin-based chemotherapy regimen (96.8%).

**Table 1:** Sociodemographic, clinical, and lifestyle characteristics of oxaliplatin-treated colorectal cancer patients at 6 weeks post-treatment

	Total population *n* = 124	Men *n* = 81 (65.3%)	Women *n* = 43 (34.7%)
**Age, mean (SD)**	63.6 (7.7)	64.0 (8.0)	62.8 (7.2)
**Smoking status, n (%)**			
Never	38 (31.4)	23 (29.1)	15 (35.7)
Former	72 (59.5)	49 (62.0)	23 (54.8)
Current	11 (9.1)	7 (8.9)	4 (9.5)
**Alcohol intake (g/day), median (IQR)**	5.7 (14.5)	9.3 (20.2)	1.7 (9.0)
**BMI (kg/m2), mean (SD), n (%)**	28.2 (4.8)	28.6 (4.9)	27.4 (4.5)
Underweight	0 (0)	0 (0)	0 (0)
Normal weight	35 (28.2)	23 (28.4)	12 (27.9)
Overweight	49 (39.5)	29 (35.8)	20 (46.5)
Obese	40 (32.3)	29 (35.8)	11 (25.6)
**Comorbidities, n (%)**			
0	39 (31.7)	29 (35.8)	10 (23.8)
1	32 (26.0)	21 (25.9)	11 (26.2)
≥ 2	52 (42.3)	31 (38.3)	21 (50.0)
**Level of Education, n (%)**			
Low	27 (21.8)	13 (16.0)	14 (32.6)
Medium	48 (38.7)	28 (34.6)	20 (46.5)
High	49 (39.5)	40 (49.4)	9 (20.9)
**Type of cancer, n (%)**			
Colon cancer	85 (68.5)	52 (64.2)	33 (76.7)
Rectal cancer	39 (31.5)	29 (35.8)	10 (23.3)
**Cancer stage, n (%)**			
I	0 (0)	0 (0)	0 (0)
II	11 (8.9)	7 (8.6)	4 (9.3)
III	113 (91.1)	74 (91.4)	39 (90.7)
**Chemotherapy regimen, n (%)**			
Capecitabine + oxaliplatin (CAPOX)	120 (96.8)	79 (97.5)	41 (95.3)
5FU + oxaliplatin (FOLFOX)	1 (0.8)	1 (1.2)	0 (0)
Else	3 (2.4)	1 (1.2)	2 (4.7)
**Chemotherapy treatment, n (%)**			
Neo-adjuvant	1 (0.8)	0 (0)	1 (2.3)
Adjuvant	123 (99.2)	81 (100)	42 (97.7)

### Change in peripheral neuropathy scores over time

**Fig. 1:**
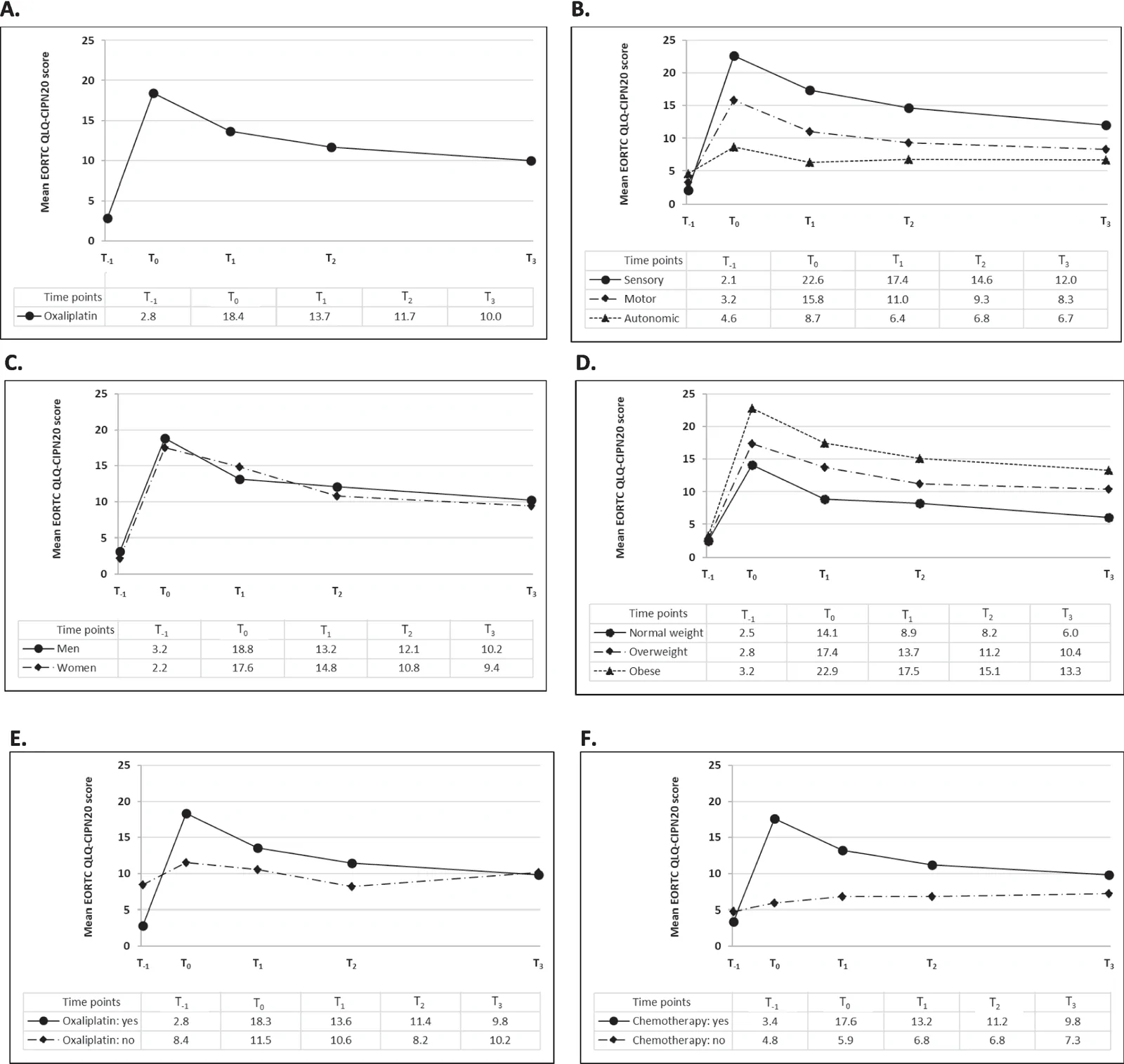
Changes over time in mean chemotherapy-induced peripheral neuropathy (CIPN) scores from diagnosis until 24 months post-treatment among colorectal cancer patients. Mean CIPN scores are shown at diagnosis (T_−1_) and at 6 weeks (T_0_), 6 months (T_1_), 12 months (T_2_), and 24 months (T_3_) post-treatment. Total CIPN scores (**A**) and sensory, motor, and autonomic CIPN symptom scores (**B**) are shown among oxaliplatin-treated patients (n = 141), with total scores also stratified for sex (**C**) and BMI categories (**D**). Additionally, differences in CIPN scores over time between patients treated with oxaliplatin-based chemotherapy (n = 141) vs. no oxaliplatin-based chemotherapy (n = 18, capecitabine monotherapy) (**E**) are shown, as well as between patients treated with chemotherapy, both oxaliplatin-based and not oxaliplatin-based (n = 159), vs. not treated with chemotherapy (n = 408) (**F**)

In oxaliplatin-treated patients (Fig. 1A), CIPN scores were lowest at diagnosis (mean: 2.8, SD: 4.1), peaked at 6 weeks post-treatment (mean: 18.4, SD: 13.0), and then gradually declined but remained on average above CIPN scores at diagnosis up until 2 years post-treatment. When comparing scores across EORTC QLQ-CIPN20 subscales (Fig. 1B), sensory subscale scores were highest at all post-treatment time points, followed by scores on the motor and autonomic subscales. No large sex differences were observed regarding changes in CIPN scores over time (Fig. 1C). Interestingly, post-treatment CIPN scores were consistently highest among participants who were obese at diagnosis and lowest in those with normal weight (Fig. 1D). All observed patterns persisted through the 60-month post-treatment follow-up (Supplementary Fig. 2).

### Longitudinal associations of CIPN with psychological distress and health-related quality of life

Results of longitudinal analyses from 6 weeks until 2 years post-treatment are presented in Table 2, showing that in fully adjusted models higher total CIPN scores were statistically significantly associated with most outcomes (HADS and EORTC QLQ-C30 scores). More specifically, higher total CIPN scores were longitudinally associated with increased levels of psychological distress (β per 5-point increase in CIPN score: 0.39; 95% CI: 0.13, 0.65), including both increased anxiety (β: 0.19; 95% CI: 0.06, 0.33) and depressive symptoms (β: 0.24; 95% CI: 0.11, 0.36). Additionally, higher total CIPN scores were longitudinally associated with decreased global health/QoL (β: −1.66; 95% CI: −2.36, −0.96), physical functioning (β: −2.26; 95% CI: −2.91, −1.60), and social functioning (β: −1.53; 95% CI: −2.48, −0.57), and with increased fatigue scores (β: 1.19; 95% CI: 0.27, 2.11). Similar patterns were observed across sensory, motor and autonomic CIPN subscales. Hybrid models showed significant intra- and inter-individual associations, with stronger inter-individual associations (Table 2). For example, a 5-point between-person CIPN difference was associated with higher psychological distress (β: 0.57; 95% CI: 0.13, 1.01) and lower global health/QoL (β: −1.72; 95% CI: −2.68, −0.76), while 5-point within-person CIPN changes showed similar yet slightly weaker associations for psychological distress (β: 0.37; 95% CI: 0.10, 0.64) and global health/QoL (β: −1.58; 95% CI: −2.62, −0.55). Comparable patterns were observed for the CIPN subscales. Notably, while no statistically significant associations were observed for the emotional and cognitive functioning subscales (EORTC QLQ-C30) in the overall models, inter-individual associations were significant in hybrid models. Analyses up to 60 months post-treatment yielded similar results (Supplementary Table 1). Overall, no clear patterns of statistical interactions were observed (data not shown).

**Table 2:** Longitudinal associations of chemotherapy-induced peripheral neuropathy (CIPN) with psychological distress and health-related quality of life (HRQoL) in 124 colorectal cancer patients followed up from 6 weeks to 2 years post-treatment

	HADS			EORTC QLQ-C30					
	psychological distress (0–42)	anxiety symptoms (0–21)	depression symptoms (0–21)	global health/QoL (0–100)	physical functioning (0–100)	social functioning (0–100)	emotional functioning (0–100)	cognitive functioning (0–100)	fatigue symptoms (0–100)
	β (95% CI)	β (95% CI)	β (95% CI)	β (95% CI)	β (95% CI)	β (95% CI)	β (95% CI)	β (95% CI)	β (95% CI)
EORTC QLQ-CIPN20 total score^4^									
Fully adjusted^1^ Intra-individual^2^ Inter-individual^3^	**0.39 **(0.13, 0.65) **0.37** (0.10, 0.64) **0.57** (0.13, 1.01)	**0.19 **(0.06, 0.33) 0.15 (−0.02, 0.31) **0.30** (0.05, 0.55)	**0.24 **(0.11, 0.36) **0.22** (0.07, 0.37) **0.27** (0.06, 0.49)	**−1.66 **(−2.36, −0.96) **−1.58** (−2.62, −0.55) **−1.72** (−2.68, −0.76)	**−2.26 **(−2.91, −1.60) **−1.97** (−2.78, −1.16) **−2.79** (−3.90, −1.69)	**−1.53 **(−2.48, −0.57) **−1.57** (−2.93, −0.21) **−1.93** (−2.96, −0.91)	−0.62 (−1.58, 0.34) −0.62 (−1.63, 0.39) **−1.45** (−2.55, −0.34)	−0.39 (−1.33, 0.55) 0.41 (−0.61, 1.42) **−1.81** (−2.93, −0.70)	**1.19 **(0.27, 2.11) 0.91 (−0.22, 2.04) **2.01** (0.87, 3.14)
EORTC QLQ-CIPN20 sensory score^4^									
Fully adjusted^1^ Intra-individual^2^ Inter-individual^3^	**0.27 **(0.10, 0.45) **0.23** (0.03, 0.44) **0.38** (0.05, 0.72)	**0.13 **(0.02, 0.23) 0.10 (−0.02, 0.23) **0.19** (0.00, 0.38)	**0.15 **(0.06, 0.24) **0.13** (0.01, 0.24) **0.19** (0.03, 0.36)	**−1.06** (−1.59, −0.53) −0.75 (−1.54, 0.05) **−1.32** (−2.04, −0.60)	**−1.52 **(−2.02, −1.02) **−1.23** (−1.85, −0.60) **−2.04** (−2.89, −1.20)	**−1.04 **(−1.77, −0.31) −0.63 (−1.67, 0.40) **−1.46** (−2.24, −0.68)	−0.45 (−1.15, −0.24) −0.34 (−1.11, 0.42) **−1.00** (−1.84, −0.15)	−0.41 (−1.13, 0.32) 0.13 (−0.65, 0.90) **−1.26** (−2.11, −0.42)	0.68 (−0.01, 1.37) 0.37 (−0.49, 1.23) **1.30** (0.43, 2.18)
EORTC QLQ-CIPN20 autonomic score^4^									
Fully adjusted^1^ Intra-individual^2^ Inter-individual^3^	**0.33 **(0.07, 0.58) **0.34** (0.15, 0.54) **1.29** (0.71, 1.88)	0.13 (−0.03, 0.29) **0.13** (0.01, 0.25) **0.74** (0.41, 1.07)	**0.21 **(0.08, 0.34) **0.22** (0.12, 0.33) **0.55** (0.25, 0.84)	**−1.07 **(−1.74, −0.39) **−1.00** (−1.75, −0.24) −1.34 (−2.79, 0.11)	**−0.80 **(−1.38, −0.23) **−0.81** (−1.41, −0.20) −0.77 (−2.60, 1.06)	−0.97 (−2.13, 0.18) **−1.15** (−2.13, −0.16) −1.52 (−3.10, 0.05)	**−0.94 **(−2.13, 0.18) **−1.31** (−2.02, −0.60) **−2.89** (−4.40, −1.38)	−0.20 (−0.88, 0.49) 0.37 (−0.37, 1.10) **−3.25** (−4.76, −1.73)	**0.96 **(0.03, 1.89) **0.87** (0.05, 1.69) **2.46** (0.82, 4.11)
EORTC QLQ-CIPN20 motor score^4^									
Fully adjusted^1^ Intra-individual^2^ Inter-individual^3^	**0.23 **(0.02, 0.44) 0.17 (−0.06, 0.41) 0.46 (0.00, 0.92)	0.10 (−0.02, 0.23) 0.06 (−0.08, 0.20) 0.25 (−0.01, 0.51)	**0.13 **(0.02, 0.25) 0.11 (−0.02, 0.24) 0.21 (−0.02, 0.43)	**−1.36 **(−2.03, −0.69) **–1.35** (−2.23, −0.47) −1.38 (−2.41, −0.34)	**−1.67 **(−2.28, −1.06) **−1.34** (−2.04, −0.63) **−2.63** (−3.81, −1.45)	**−1.53 **(−2.33, −0.74) **−1.45** (−2.61, −0.29) **−1.61** (−2.71, −0.51)	−0.46 (−1.15, 0.23) −0.09 (−0.95, 0.77) −1.16 (−2.33, 0.02)	−0.24 (−0.95, 0.47) 0.39 (−0.48, 1.25) **−1.45** (−2.63, −0.26)	**1.27 **(0.52, 2.02) 0.77 (−0.20, 1.73) **2.04** (0.86, 3.23)

EORTC QLQ-C30: European Organization for Research and Treatment of Cancer Quality of Life Questionnaire-Core 30; HADS: Hospital Anxiety and Depression Scale; β: Beta (i.e., regression coefficient); EORTC QLQ-CIPN20: European Organization for Research and Treatment of Cancer Quality of Life Questionnaire-Chemotherapy-Induced Peripheral Neuropathy 20; CI: confidence interval

^1^Linear mixed regression model, adjusted for time since end of treatment, sex, age, smoking status, alcohol intake, BMI, number of comorbidities, tumor stage, and CIPN score at diagnosis. Beta coefficients indicate the average change in outcome score over time for every 5-point increase in CIPN score

^2^Hybrid linear mixed regression model including intra-individual differences (adjusted for the same confounders as mentioned above). Beta coefficients indicate the average change in outcome scores over time for every 5-point increase in CIPN score within individuals

^3^Hybrid linear mixed regression model including inter-individual differences (adjusted for the same confounders as mentioned above). Beta coefficients indicate the average difference in outcome scores over time for every 5-point difference in CIPN score between individuals

^4^Random slopes were added, based on significant likelihood ratio tests, to models for total CIPN score with HADS psychological distress, and EORTC QLQ-C30 social, emotional, and cognitive functioning, and fatigue; for sensory CIPN score with HADS depression, and EORTC QLQ-C30 social, emotional, and cognitive functioning, and fatigue; for autonomic CIPN score with HADS psychological distress, anxiety, and depression, and EORTC QLQ-C30 social and emotional functioning, and fatigue; and for motor CIPN score with HADS anxiety, and EORTC QLQ-C30 global health/QoL, emotional and cognitive functioning, and fatigue

Statistically significant results (P < 0.05) are indicated in bold

### Differences in CIPN scores over time between treatment groups

Oxaliplatin-treated patients exhibited almost twofold higher CIPN scores on average at 6 weeks post-treatment than patients not treated with oxaliplatin-based chemotherapy (Fig. 1E). Thereafter, CIPN scores in oxaliplatin-treated patients gradually converged with scores of non-oxaliplatin-treated patients until the 2-year time point (Fig. 1E). Of note, compared to oxaliplatin-treated patients, those not receiving oxaliplatin-based chemotherapy but capecitabine monotherapy had somewhat higher CIPN scores at diagnosis, which remained relatively stable over time. Compared to patients not receiving chemotherapy, consistently higher CIPN scores were observed among chemotherapy-treated patients, who had threefold higher scores at 6 weeks post-treatment (Fig. 1F). As shown in Table 3, treatment with an oxaliplatin-based chemotherapy regimen was statistically significantly associated with on average higher total CIPN scores between 6 weeks and 2 years post-treatment (β: 5.17; 95% CI: 0.90, 9.44), which was attributable to higher sensory CIPN subscale scores (β: 10.58; 95% CI: 5.46, 15.70). Chemotherapy treatment in general was longitudinally associated with higher total CIPN scores (β: 7.92; 95% CI: 5.93, 9.92), driven mainly by sensory CIPN symptoms (β: 10.55; 95% CI: 7.29, 13.81) and to a lesser extent by autonomic (β: 3.12; 95% CI: 0.74, 5.50) and motor CIPN symptoms (β: 5.75; 95% CI: 3.44, 8.06). These findings remained consistent up to 60 months post-treatment (Supplementary Fig. 2 and Table 2). None of the interaction models were statistically significant (data not shown).

**Table 3:** Longitudinal associations of the type of chemotherapy treatment received with chemotherapy-induced peripheral neuropathy (CIPN) scores, as well as of chemotherapy treatment versus no chemotherapy treatment with CIPN scores in colorectal cancer patients followed up from 6 weeks to 2 years post-treatment

	EORTC QLQ-CIPN20			
	total score (0–100)	sensory score (0–100)	autonomic score (0–100)	motor score (0–100)
	β (95% CI)	β (95% CI)	β (95% CI)	β (95% CI)
Oxaliplatin-based chemotherapy treatment vs. non-oxaliplatin-based chemotherapy treatment (ref.)^2^				
Fully adjusted^1^	**5.17** (0.90, 9.44)	**10.58** (5.46, 15.70)	−0.76 (−6.93, 5.42)	0.72 (−6.53, 7.97)
Chemotherapy treatment vs. no chemotherapy treatment (ref.)^3^				
Fully adjusted^1^	**7.92** (5.93, 9.92)	**10.55** (7.29, 13.81)	**3.12** (0.74, 5.50)	**5.75** (3.44, 8.06)

EORTC QLQ-CIPN20: European Organization for Research and Treatment of Cancer Quality of Life Questionnaire-Chemotherapy-Induced Peripheral Neuropathy 20; β: Beta (i.e., regression coefficient); CI: confidence interval

^1^Linear mixed regression model, adjusted for tumor stage and BMI, number of comorbidities and CIPN score at diagnosis. Beta coefficients indicate the average difference in CIPN scores over time between groups defined based on type of chemotherapy treatment

^2^Random slopes were added, based on significant likelihood ratio tests, to models with total and sensory CIPN scores

^3^Random slopes were added, based on significant likelihood ratio tests, to models with sensory CIPN scores

Statistically significant results (*P* < 0.05) are indicated in bold

## Discussion

This study examined how CIPN symptoms relate to psychological distress and HRQoL in CRC survivors up to 5 years post-treatment. Higher CIPN levels over time were associated with greater psychological distress (including symptoms of anxiety and depression), lower global health/QoL, lower physical and social functioning, and more fatigue symptoms. Both intra-individual and inter-individual associations were observed, with stronger effects between individuals suggesting that those with persistently more severe CIPN symptoms report worse outcomes. Comparatively, albeit of a lesser magnitude, intra-individual associations nonetheless indicated that increases in CIPN symptoms within individuals over time were associated with increases in psychological distress and decreases in HRQoL. Though CIPN symptoms declined over time after treatment, they remained above pre-treatment levels, with sensory symptoms consistently being the highest on average, and continued to negatively impact psychological distress and HRQoL in CRC survivors up to 5 years post-treatment. No differences in CIPN symptoms between men and women were observed on average over time. Interestingly, oxaliplatin-treated patients with higher BMI reported on average more severe symptoms over time. As a secondary aim, the influence of chemotherapy and the specific chemotherapy regimen on CIPN over time was explored. As expected, CRC patients treated with chemotherapy reported higher levels of CIPN symptoms up to 5 years post-treatment compared to patients not receiving chemotherapy. An oxaliplatin-based chemotherapy regimen was longitudinally associated with higher CIPN levels, especially sensory symptoms, confirming that chemotherapy, and particularly oxaliplatin, is a key driver of CIPN.

Studies on CIPN, psychological distress and HRQoL in CRC survivors remain limited. In a cross-sectional study among CRC survivors 2–11 years post-diagnosis, Mols et al. found that CRC survivors with higher scores on the sensory subscale of the EORTC QLQ-CIPN20 reported lower functioning and global health/QoL compared to those with lower scores [26]. Similarly, results from a prospective cohort study by Bonhof et al. showed that sensory and motor CIPN symptoms up to two years post-diagnosis were related to poorer global health/QoL, as well as lower physical, emotional, cognitive, and social functioning among CRC patients, with small to medium clinically relevant effect sizes observed for most of the subscale scores [27]. A cross-sectional study by Hong et al. found significant associations of CIPN symptoms with both anxiety and depression among cancer patients, including CRC, indicating that CIPN may be a risk factor for these symptoms of psychological distress [28]. The above findings align with ours. Additionally, in line with our secondary aims, Mols et al. reported higher sensory CIPN scores in colon cancer patients treated with chemotherapy compared to those not receiving chemotherapy. Patients undergoing oxaliplatin-based treatment similarly showed higher sensory CIPN scores than those on non-oxaliplatin-based treatment regimens. Moreover, similar comparisons in rectal cancer patients revealed significantly higher scores on both the sensory and motor subscales [26]. We observed that low-level CIPN-like symptoms were consistently reported on average by the group of CRC patients not treated with chemotherapy. This might be explained by several reasons, such as similar symptoms being related to comorbid conditions (e.g., diabetes or chronic obstructive pulmonary disease) as well as the non-specific nature of items in the EORTC QLQ-CIPN20 (e.g., “Did you have tingling fingers or hands?”).

In terms of clinical relevance, the observed longitudinal associations (Table 2) may seem to fall short of minimal clinically important differences (MCID) for the HADS (1.7 points) and EORTC QLQ-C30 (≥ 10 points) [29, 30]. This does not mean, however, that the longitudinal associations of higher levels of CIPN symptoms with increased psychological distress and decreased HRQoL can be dismissed. Longitudinal associations were modelled per 5-point increase in CIPN scores, yet the impact of chemotherapy treatment on CIPN may be even larger in clinical practice, especially in the immediate post-treatment period (Fig. 1) and particularly sensory symptoms (Table 3). Our findings thus indicate that many patients treated with oxaliplatin experience long-lasting CIPN symptoms up to 5 years post-treatment, negatively impacting psychological and physical well-being. Therefore, early identification of high-risk patients, careful monitoring of CIPN, and timely application of interventions aimed at preventing or mitigating CIPN symptoms must be part of peri-treatment care and aftercare following oxaliplatin-based chemotherapy in CRC patients. Clinical prediction models to estimate the risk of developing (chronic) CIPN based on clinical and patient characteristics, such as BMI or other potential predictive factors, could be of value for this purpose, yet such models are not available for CRC patients. Currently, few effective treatments or preventive options exist for CIPN. Duloxetine is the primary treatment method for neuropathic pain [31]; however, the American Society of Clinical Oncology has suggested gabapentin, pregabalin, nortriptyline, amitriptyline, and a few topical compounds as reasonable alternatives [32]. A notable alternative is mangafodipir, which is a cytoprotectant capable of preventing or alleviating oxaliplatin-induced peripheral neuropathy [13, 33]. Despite these options, the lack of robust clinical trials highlights the need to consider various patient factors and symptom coexistence in treatment decisions. Non-pharmacological interventions, such as dietary supplements, post-treatment exercise, and moderate cryotherapy during treatment, have shown potential for reducing or preventing CIPN [33–37]. As at present there appears to be no consensus and standardization on which intervention to use in practice, more research on interventions for CRC patients experiencing chronic CIPN is warranted.

A strength of this study was its prospective design, featuring repeated measurements of CIPN and the main outcome variables, as well as relevant confounders. Even so, a limitation of the current study is its observational nature, limiting our ability to draw causal conclusions about observed associations. Additionally, there may have been selective responses at baseline, as patients with higher psychological distress and lower HRQoL at diagnosis might have been reluctant to join the study, potentially underestimating associations. A further limitation may be an increased chance of false positive findings due to multiple testing, even though the observed pattern of statistically significant findings were in line with our a priori hypotheses. And finally, the sample size for our secondary analyses, examining the influence of the type of chemotherapy treatment, was limited. Especially the very small number of patients not treated with an oxaliplatin derivative reduced our study's power and ability to adjust for confounders. Results of these secondary analyses should be interpreted with caution.

In conclusion, our longitudinal findings suggest that persisting CIPN symptoms among oxaliplatin-treated CRC patients have a long-lasting impact on psychological distress and HRQoL up to 5 years post-treatment. Future studies should confirm relations between the type of chemotherapy and post-treatment CIPN symptoms in CRC patients, and explore effects of dose reductions or switching to alternative therapies, ideally using real-world data that is generalizable to clinical practice. Moreover, more research is needed on the potential of non-pharmacological approaches (e.g., lifestyle interventions) as valuable avenues to alleviate CIPN symptoms and help patients to better self-manage their symptoms.

## Supplementary Information

Below is the link to the electronic supplementary material.

Supplementary file1 (DOCX 226 kb)

## Data Availability

Data described in the manuscript and analytic code will be made available upon reasonable request, to be sent to Dr. Martijn Bours, Department of Epidemiology, GROW Research Institute for Oncology and Reproduction, Maastricht University, The Netherlands (email: m.bours@maastrichtuniversity.nl).
