# Supplementary material for: Longitudinal associations of chemotherapy-induced peripheral neuropathy with psychological distress and health-related quality of life in colorectal cancer survivors: a prospective cohort study
Source: J Cancer Surviv. 2025 Nov 21;20(5):2339–49. doi: 10.1007/s11764-025-01906-1 (PMC13723573; doi:10.1007/s11764-025-01906-1)
Supplement: Supplementary file 1 [file 11764_2025_1906_MOESM1_ESM.docx]

# Supplementary Information

Non-eligible persons (n=63)

Does not meet inclusion criteria (n=61)

Person died (n=2)

Persons asked to participate

*n = 1411*

Eligible persons

*n = 1348*

Persons not wanting to participate (n=750)

Too burdensome next to regular care (n=308)

Emotionally too burdensome (n=178)

Personal circumstances (n=31)

Takes too much time (n=60)

Change in treatment policy (n=20)

Event in family (n=2)

Other/unknown (n=108)

No specific reason (n=43)

Persons signed informed consent

*n = 598*

Non-eligible persons (n=31)

In situ tumor/no carcinoma (n=7)

Stage IV tumor (n=12)

Treatment in other hospital (n=8)

Different type of tumor (n=2)

Treatment already started (n=2)

Persons lost to follow-up among oxaliplatin-treated participants

Persons included **at diagnosis**

*n = 567, 43%^1^*

***n = 141 persons treated with oxaliplatin***

Persons lost to follow-up prior to 6-week time point (n=15)

Emotionally too burdensome (n=5)

Personal circumstances (n=2)

Takes too much time (n=1)

Person died (n=2)

Event in family (n=2)

Other (n=3)

*Persons without 6 weeks visit (n=9)*

Persons included **at 6 weeks**

*n = 504, 92%^2^*

***n = 124 persons treated with oxaliplatin***

Persons lost to follow-up prior to 6-month time point (n=13)

Emotionally too burdensome (n=6)

Personal circumstances (n=2)

Takes too much time (n=1)

Person died (n=1)

Event in family (n=1)

Other (n=2)

*Persons without 6 months visit (n=24)*

Persons included **at** **6 months**

*n = 452, 93%^2^ (incl. n=9 who missed 6 weeks visit)*

***n = 111 persons treated with oxaliplatin***

*Persons without 12 months visit (n=8)*

Persons lost to follow-up prior to 12-month time point (n=6)

Emotionally too burdensome (n=1)

Personal circumstances (n=4)

Takes too much time (n=1)

Persons included **at** **12 months**

*n = 435, 94%^2^ (incl. n=21^3^ who missed 6 months visit)*

***n = 106 persons treated with oxaliplatin***

Persons lost to follow-up prior to 24-month time point (n=8)

Personal circumstances (n=3)

Takes too much time (n=1)

Person died (n=4)

*No visit before October 1st 2021 (n=5)*

*Persons without 24 months visit (n=10)*

Persons included **at** **24 months**

*n = 382, 96%^2^ (incl. n=6 who missed 12 months visit)*

***n = 93 persons treated with oxaliplatin***

Persons lost to follow-up prior to 60-month time point (n=21)

No consent for future participation (n=6)

Emotionally too burdensome (n=4)

Personal circumstances (n=3)

Takes too much time (n=1)

Person died (n=4)

Event in family (n=1)

Other (n=2)

*No visit before October 1st 2021 (n=162)*

Persons included **at 60 months**

*n = 131, 63%^2^*

***n = 35 persons treated with oxaliplatin***

**Supplementary Figure 1.** Flow diagram of study participants included and followed up between April 2012 and October 2021, including the subsample of participants treated with oxaliplatin-based chemotherapy. ^1^ Response rate at inclusion = persons signed informed consent / (persons asked – non-eligible persons). ^2^ Response rate during follow-up = (persons with home visits) / (persons with home visits + persons lost to follow-up – persons died). The declining number of participants at subsequent time points during post-treatment follow-up is mainly due to the fact that not all participants included at diagnosis from April 2012 onwards had reached these time points in October 2021. ^3^ Of the 24 participants without a 6-month visit, three participants did not have a 12-month visit. Of the 8 participants without a 12-month visit, two participants did not have a 24-month visit.

| A.  | B.  |
| --- | --- |
| C.  | **D.**  |
| E.  | **F.**  |

**Supplementary Figure 2.** Changes over time in mean chemotherapy-induced peripheral neuropathy (CIPN) scores from diagnosis until 60 months post-treatment among colorectal cancer patients. Mean CIPN scores are shown at diagnosis (T_-1_) and at 6 weeks (T_0_), 6 months (T_1_), 12 months (T_2_), 24 months (T_3_), and 60 months (T_4_) post-treatment. Total CIPN scores (**A**) and sensory, motor, and autonomic CIPN symptom scores (**B**) are shown among oxaliplatin-treated patients (n=141), with total scores also stratified for sex (**C**) and BMI categories (**D**). Additionally, differences in CIPN scores over time between patients treated with oxaliplatin-based chemotherapy vs. no oxaliplatin-based chemotherapy (n=18) (**E**) are shown, as well as between patients treated with chemotherapy, both oxaliplatin-based and not oxaliplatin-based (n=159), vs. not treated with chemotherapy (n=408) (**F**).

**Supplementary Table 1.** Longitudinal associations of chemotherapy-induced peripheral neuropathy (CIPN) scores with psychological distress and health-related quality of life (HRQoL) in 124 colorectal cancer patients followed up from 6 weeks to 5 years post-treatment.

|  | HADS | | | EORTC QLQ-C30 | | | | | |
| --- | --- | --- | --- | --- | --- | --- | --- | --- | --- |
|  | **psychological distress**  (0-42) | **anxiety symptoms**  (0-21) | **depression symptoms**  (0-21) | **global health/QoL**  (0-100) | **physical functioning**  (0-100) | **social functioning**  (0-100) | **emotional functioning**  (0-100) | **cognitive functioning**  (0-100) | **fatigue symptoms**  (0-100) |
|  | β (95% CI) | β (95% CI) | β (95% CI) | β (95% CI) | β (95% CI) | β (95% CI) | β (95% CI) | β (95% CI) | β (95% CI) |
| EORTC QLQ-CIPN20 total score^4^ | | | | | | | | | |
| Fully adjusted^1^  Intra-individual^2^  Inter-individual^3^ | **0.46**  (0.24, 0.68)  **0.43**  (0.18, 0.68)  **0.54**  (0.10, 0.99) | **0.22**  (0.08, 0.35)  **0.19**  (0.03, 0.35)  **0.28**  (0.03, 0.53) | **0.24**  (0.13, 0.36)  **0.24**  (0.10, 0.37)  **0.26**  (0.05, 0.48) | **-1.67**  (-2.33, -1.00)  **-1.52**  (-2.48, -0.55)  **-1.81**  (-2.76, -0.87) | **-2.24**  (-2.87, -1.60)  **-1.94**  (-2.72, -1.16)  **-2.82**  (-3.93, -1.71) | **-1.76**  (-2.53, -1.00)  **-1.61**  (-2.86, -0.35)  **-1.86**  (-2.84, -0.87) | -**0.96**  (-1.67, 0.25)  -0.71  (-1.65, 0.23)  **-1.31**  (-2.42, -0.19) | **-0.79**  (-1.52, 0.07)  -0.10  (-1.07, 0.88)  **-1.69**  (-2.80, -0.59) | **1.63**  (0.87, 2.39)  **1.24**  (0.21, 2.28)  **2.10**  (0.96, 3.24) |
| EORTC QLQ-CIPN20 sensory score^4^ | | | | | | | | | |
| Fully adjusted^1^  Intra-individual^2^  Inter-individual^3^ | **0.29**  (0.12, 0.46)  **0.27**  (0.07, 0.46)  **0.36**  (0.03, 0.70) | **0.14**  (0.04, 0.24)  0.12  (0.00, 0.24)  **0.18**  (-0.01, 0.37) | **0.16**  (0.07, 0.24)  **0.14**  (0.04, 0.25)  **0.19**  (0.02, 0.35) | **-1.09**  (-1.60, -0.58)  **-0.75**  (-1.49, -0.01)  **-1.40**  (-2.11, -0.69) | **-1.47**  (-1.95, -0.98)  **-1.16**  (-1.76, -0.55)  **-2.07**  (-2.91, -1.22) | **-1.16**  (-1.74, -0.58)  -0.73  (-1.70, 0.23)  **-1.42**  (-2.17, -0.67) | -0.61  (-1.15, -0.07)  -0.39  (-1.11, 0.32)  **-0.90**  (-1.75, -0.05) | -0.63  (-1.18, 0.08)  -0.21  (-0.95, 0.53)  **-1.16**  (-2.01, -0.32) | 0.97  (0.39, 1.55)  0.64  (-0.15, 1.44)  **1.36**  (0.48, 2.25) |
| EORTC QLQ-CIPN20 autonomic score^4^ | | | | | | | | | |
| Fully adjusted^1^  Intra-individual^2^  Inter-individual^3^ | **0.42**  (0.25, 0.59)  **0.35**  (0.18, 0.53)  **1.27**  (0.68, 1.86) | **0.21**  (0.10, 0.31)  **0.15**  (0.04, 0.26)  **0.73**  (0.40, 1.05) | **0.23**  (0.14, 0.32)  **0.20**  (0.11, 0.30)  **0.54**  (0.25, 0.84) | **-0.90**  (-1.53, -0.27)  **-0.81**  (-1.50, -0.11)  -1.31  (-2.76, 0.14) | **-0.63**  (-1.18, -0.08)  **-0.63**  (-1.21, -0.06)  -0.64  (-2.48, 1.19) | **-1.08**  (-1.87, -0.30)  **-0.97**  (-1.88, -0.07)  -1.40  (-2.93, 0.12) | **-1.43**  (-2.04, 0.83)  **-1.19**  (-1.84, -0.54)  **-2.87**  (-4.39, -1.35) | -0.29  (-0.93, 0.36)  0.21  (-0.48, 0.91)  **-3.26**  (-4.76, -1.77) | **1.17**  (0.48, 1.85)  **0.92**  (0.18, 1.66)  **2.54**  (0.87, 4.21) |
| EORTC QLQ-CIPN20 motor score^4^ | | | | | | | | | |
| Fully adjusted^1^  Intra-individual^2^  Inter-individual^3^ | **0.27**  (0.07, 0.47)  **0.24**  (0.01, 0.46)  0.44  (-0.03, 0.90) | **0.13**  (0.01, 0.25)  0.10  (-0.04, 0.24)  0.24  (-0.02, 0.50) | **0.15**  (0.04, 0.25)  **0.13**  (0.01, 0.25)  0.20  (-0.03, 0.43) | **-1.39**  (-2.04, -0.74)  **-1.35**  (-2.19, -0.51)  **-1.45**  (-2.48, -0.43) | **-1.84**  (-2.43, -1.24)  **-1.55**  (-2.23, -0.86)  **-2.71**  (-3.89, -1.53) | **-1.51**  (-2.27, -0.75)  **-1.49**  (-2.59, -0.39)  **-1.53**  (-2.60, -0.46) | -0.48  (-1.15, 0.19)  -0.22  (-1.04, 0.60)  -1.02  (-2.20, 0.17) | -0.43  (-1.12, 0.26)  0.04  (-0.81, 0.89)  **-1.35**  (-2.53, -0.18) | **1.39**  (0.67, 2.11)  **0.96**  (0.06, 1.87)  **2.15**  (0.96, 3.34) |

Abbreviations: EORTC QLQ-C30: European Organization for Research and Treatment of Cancer Quality of Life Questionnaire - Core 30; HADS: Hospital Anxiety and Depression Scale; β: Beta (i.e., regression coefficient); EORTC QLQ-CIPN20: European Organization for Research and Treatment of Cancer Quality of Life Questionnaire - Chemotherapy-Induced Peripheral Neuropathy 20; CI: confidence interval.

^1^Linear mixed regression model, adjusted for time since end of treatment, sex, age, smoking status, alcohol intake, BMI, number of comorbidities, tumor stage, and CIPN score at diagnosis. Beta coefficients indicate the average change in outcome score over time for every 5-point increase in CIPN score.

^2^Hybrid linear mixed regression model including intra-individual differences (adjusted for the same confounders as mentioned above). Beta coefficients indicate the average change in outcome scores over time for every 5-point increase in CIPN score within individuals.

^3^Hybrid linear mixed regression model including inter-individual differences (adjusted for the same confounders as mentioned above). Beta coefficients indicate the average difference in outcome scores over time for every 5-point difference in CIPN score between individuals.

^4^Random slopes were added, based on significant likelihood ratio tests, to models for total CIPN score with HADS psychological distress and depression, and EORTC QLQ-C30 social, emotional, and cognitive functioning, and fatigue; for sensory CIPN score with EORTC QLQ-C30 social and emotional functioning, and fatigue; for autonomic CIPN score with HADS psychological distress, anxiety, and depression, and EORTC QLQ-C30 social and emotional functioning, and fatigue; and for motor CIPN score with EORTC QLQ-C30 global health/QoL, emotional and cognitive functioning, and fatigue.

Statistically significant results (P<0.05) are indicated in bold.

**Supplementary Table 2**. Longitudinal associations of the type of chemotherapy treatment received with chemotherapy-induced peripheral neuropathy (CIPN) scores, as well as of chemotherapy treatment versus no chemotherapy treatment with CIPN scores in colorectal cancer patients followed up from 6 weeks to 5 years post-treatment.

|  | EORTC QLQ-CIPN20 | | | |
| --- | --- | --- | --- | --- |
|  | **total score**  (0-100) | **sensory score**  (0-100) | **autonomic score**  (0-100) | **motor score**  (0-100) |
|  | β (95% CI) | β (95% CI) | β (95% CI) | β (95% CI) |
| Oxaliplatin-based chemotherapy treatment vs. non-oxaliplatin-based chemotherapy treatment (ref.)^2^ | | | | |
| Fully adjusted^1^ | **5.07** (0.79, 9.36) | **10.40** (5.28, 15.52) | -0.57 (-6.79, 5.65) | 0.61 (-6.66, 7.88) |
| Chemotherapy treatment vs. no chemotherapy treatment (ref.)^3^ | | | | |
| Fully adjusted^1^ | **7.65** (5.64, 9.67) | **10.16** (6.89, 13.43) | **3.09** (0.70, 5.49) | **5.54** (3.22, 7.86) |

Abbreviations: EORTC QLQ-CIPN20: European Organization for Research and Treatment of Cancer Quality of Life Questionnaire - Chemotherapy-Induced Peripheral Neuropathy 20; β: Beta (i.e., regression coefficient); CI: confidence interval.

^1^Linear mixed regression model, adjusted for tumor stage and BMI, number of comorbidities and CIPN score at diagnosis. Beta coefficients indicate the average difference in CIPN scores over time between groups defined based on type of chemotherapy treatment.

^2^Random slopes were added, based on significant likelihood ratio tests, to models with total and sensory CIPN scores.

^3^Random slopes were added, based on significant likelihood ratio tests, to models with sensory CIPN scores.

Statistically significant results (P<0.05) are indicated in bold.
